# A crystal plasticity model for metal matrix composites considering thermal mismatch stress induced dislocations and twins

**DOI:** 10.1038/s41598-021-95439-z

**Published:** 2021-08-06

**Authors:** Y. N. Hou, K. M. Yang, J. Song, H. Wang, Y. Liu, T. X. Fan

**Affiliations:** 1 State Key Laboratory of Metal Matrix Composites, School of Materials Science and Engineering, Shanghai Jiao Tong University, Shanghai, 200240 China; 2 State Key Laboratory of Mechanical System and Vibration, Shanghai Jiao Tong University, Shanghai, 200240 China

**Keywords:** Materials science, Nanoscience and technology

## Abstract

Originated at heterogeneous interfaces with distinct coefficient of thermal expansion (CTE), thermal mismatch stress is one of the critical influential factors to mechanical properties of metal matrix composites (MMCs). This stress is normally accommodated plastically by various defects, for example, high-density dislocations and twins in Al near heterogeneous interfaces in SiC/Al composites. Basic knowledge on the influence of defect characteristics is important but difficult to extrapolate from experimental results. However, existed theoretical models more focus on the influence of dislocation density, but less focus on defects variety, volume and distribution. In this paper, we propose a physics-based crystal plasticity model that has the capability of dealing with thermal mismatch stress induced dislocations and twins (denoted as TMDT model). The proposed TMDT model that is implemented in the Visco-Plastic Self-Consistent (VPSC) method considers defect heterogeneous distribution (gradient range), defect type (dislocations vs. twins) and defect volume fraction (twin spacing vs. twin volume). We demonstrate the validity and the capability of the VPSC-TMDT model in SiC/Al composites with thermal mismatch induced dislocations or twins. Furthermore, this model predicts the ultra-high strength of Graphene/Copper composites with high-density nanoscale twins, which is in turn the future aim for such nanocomposites.

## Introduction

**Figure 1:**
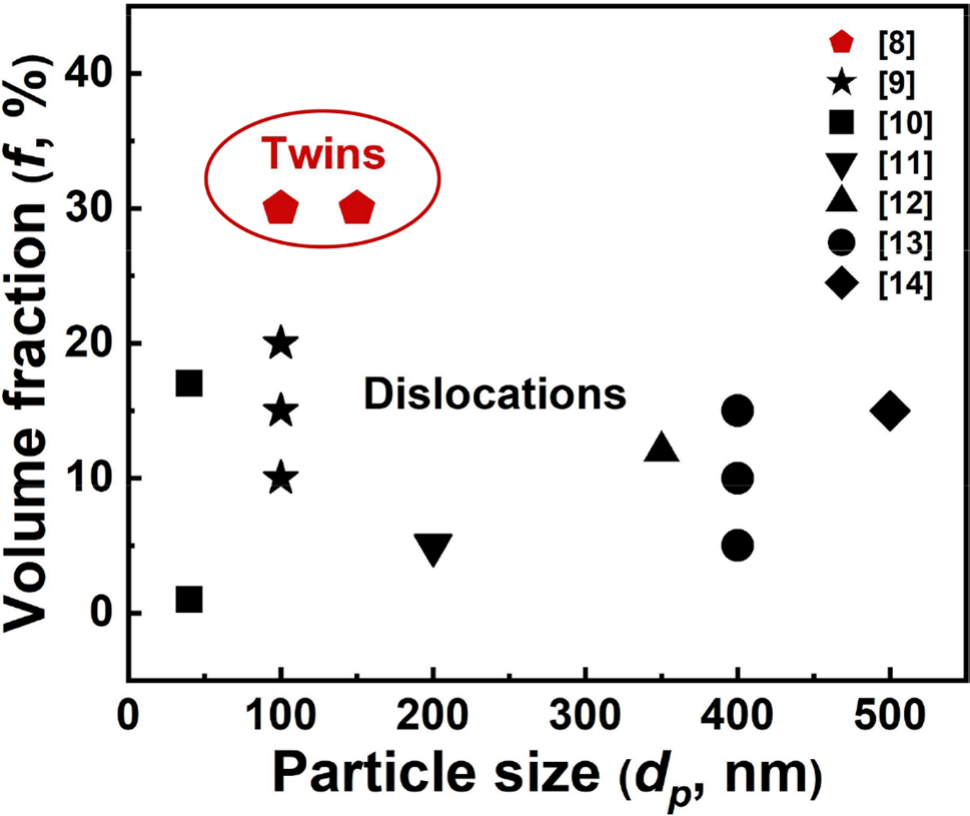
The variation of thermal mismatch defect type with the SiC volume fraction and size. For SiC/Al composites with low volume fraction and larger size of particles, the defect type in the thermal mismatch affected region is usually dislocations. On the contrary, thermal mismatch twins appear near the SiC/Al interfaces with high volume fraction and smaller size of particles.

**Figure 2:**
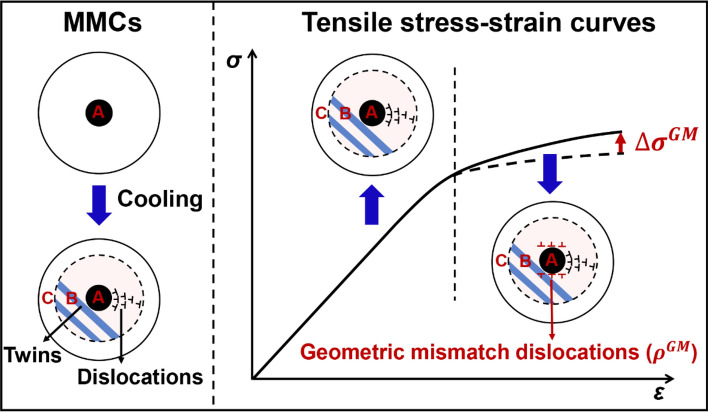
Schematic diagram showing the mechanical property of MMCs with pre-existed thermal mismatch defects. The pre-existed thermal mismatch dislocations or nanoscale twins would appear in MMCs after the cooling fabrication process. The typical grain of MMCs is comprised of reinforcement particles (region A), affected region (region B), and non-affected region (region C). The yield strength of MMCs is determined by the pre-existed defect distribution, type, and volume fraction. The flow stress during the plastic deformation is mainly influenced by the geometric mismatch dislocations (${\rho }^{GM}$) that generated near the reinforcement/ matrix heterogeneous interfaces.

Metal matrix composites (MMCs) with various kinds of macro- and nano-sized reinforcements and metal counterparts, have been increasingly demanded for the applications in the fields of aerospace, transportation and electronics etc., due to their enhanced overall performance compared to the metal matrix^1–3^. In general, thermal mismatch stress that originated at the reinforcement/matrix heterogeneous interfaces is influenced by the mismatch of coefficient of thermal expansion (CTE) between reinforcements and matrix during the heating/cooling fabrication process^4–7^. This stress is normally accommodated plastically by various defects in the matrix, such as dislocations and twins. As shown in Fig. 1, the survey of SiC/Al composite system shows that compared to thermal mismatch stress induced dislocations, the thermal mismatch stress induced nanoscale twins are highly likely to occur in composites with smaller particle size and higher volume fraction^8–14^. Since SiC particles are mostly distributed inside of the grains, there exists thermal mismatch affected region that includes those defects, and non-affected region with almost no thermal mismatch stress induced defects. Schematic in Fig. 2 illustrates the region A (reinforcement particles), region B (affected region), and region C (non-affected region) in a grain of MMCs. Both experimental and simulation results have shown that the defect type, density, and distribution range in region B could significantly influence the mechanical properties of MMCs^8,15–17^.

Since thermal mismatch stress induced dislocations and twins are pre-existed before plastic deformation, these defects would contribute to the enhancement of yield strength (${\sigma }_{y}$) of composites. The classical Taylor relation suggests the increment of ${\sigma }_{y}$ with increasing dislocation density ($\rho$)^7^. Kouzeli et al.^18^ found that, for the Al_2_O_3_/Al composites fabricated by the pressure infiltration method, the ${\sigma }_{y}$ was improved from 73 to 148 MPa when the thermal mismatch induced dislocation density (${\rho }^{CTE}$) increased from 5 × 10^12^ to 5 × 10^13^ m^−2^. In addition, a spherical dislocation punching model that considering the distribution range of dislocations was then applied, and Dong et al.^15^ concluded that when increasing the volume fraction of the particle reinforcements, the distribution range of dislocations would decrease while the ${\rho }^{CTE}$ increased, thus leading to ${\sigma }_{y}$ enhancement. Furthermore, the density and distribution range of the dislocations was then considered by extending the dislocation punching model into the finite element method (FEM) in SiC/Al composites at low deformation strain^19,20^. Therefore, it can be inferred that the mechanical properties of MMCs are closely related to both density and distribution range of the dislocations. Moreover, according to most of the microstructural characterization results^21–23^, the dislocation density actually would decrease gradually with the increment of radial distance from the heterogeneous interface, suggesting a possible gradient distribution of the dislocations. Consequently, it is important to understand the influence of gradient dislocation distribution on mechanical response of MMCs.

Apart from the dislocations, other types of defects such as nanoscale twins, have been also recently observed. Yang et al.^8^ characterized high-density nanoscale twins instead of dislocations near the interfaces of the SiC/Al composites when the volume fraction of submicron SiC particles reached 30 vol.% (as shown in Fig. 1). However, this work only focused on thermal stabilities of these nanoscale twins, the investigation on connecting twin characteristics (such as density, orientation, and spacing etc.) with mechanical properties of composites was rarely studied. One possible reason may be attributed to the co-existence of dislocations and twins in the composites, which made it difficult to explore the twinning effects. It is well known that, the twin boundary can not only block dislocation motion but also store dislocations, which benefits to enhance the strength of metals meanwhile maintaining a good plasticity^24,25^. Besides, due to the relatively low scattering strength of twin boundary on electrons and phonons, good electrical and thermal conductivities of the metals are also expected^25,26^. Therefore, understanding the influence of twinning characteristics on mechanical responses is important but difficult to extrapolate from the experiments.

The Visco-Plastic Self-Consistent (VPSC), which is famous as a mesoscopic polycrystalline method that proposed by Lebensohn and Tomé^27^, has been widely employed to explore the relationship between the microstructure and mechanical stress–strain responses of the single-phase and multi-phase metals during deformation^28–30^. Besides, different from the conventional macroscopic constitutive model, the texture evolution of each grain in the polycrystalline metals is also simulated through the VPSC^31,32^. Especially, when the twins are implanted into the grains, the relationship between the specific parameters of twins and macro mechanical properties of metals can be directly established^33^. By applying the VPSC method with predominant twin reorientation, Pan et al.^34^ confirmed that, at early shear deformation of the rolled AZ31B alloy, the mechanical discrepancy was mainly due to the activation of extension twin and prismatic slip. Through an improved VPSC method and simultaneously considering the twinning and detwinning, Wang et al.^35^ studied the effects of strain rate-sensitivity (SRS) on mechanical behavior of Mg alloys under wide range of applied strain rates. The results showed that, the rate dependent behaviors of Mg alloys could be better described by using multiple SRSs associated to each operative deformation mechanism. Apart from the effect of twining or detwinning, the interactions between slip and twinning in pure Ti were also taken into account in the polycrystal modeling by Wang et al.^36^. It was uncovered that, after the inclusion of slip-system level back stress development due to dislocation density accumulation, the predicted mechanical properties agreed well with the experimental measured results. Hence, the VPSC method provides a possibility to separately study the effect of twins on the mechanical properties of MMCs, and it is necessary to develop a modified VPSC method that considering the following three aspects (Fig. 2): (1) setup of a spherical reinforcement and heterogeneous interfaces inside a grain; (2) pre-existed dislocations and nanoscale twins before plastic deformation and their interactions and evolution during plastic deformation; (3) gradient distribution of defects with the increment of radial distance. In fact, the gradient dislocation distribution can be quantified by dividing finite subregions in region B.

In this work, we proposed a physical-based crystal plasticity model that had the capability of dealing with thermal mismatch stress induced dislocations and twins (denoted as TMDT model). Then, the mechanical responses of SiC/Al composites were predicted through the VPSC-TMDT model, with further demonstration of the validation by comparing with the experimental results. The results indicate that the proposed TMDT model is able to consider the key features associated with thermal mismatch dislocations and twins, as well as the mechanical stress–strain responses in the plastic deformation region. In addition, the proposed model was also applied to predict the mechanical properties of graphene (Gr)/copper (Cu) nanocomposites. Our findings emphasize the dominate influence of thermal mismatch induced nanoscale twins, which may require the higher volume fraction and smaller size of reinforcements.

## Constitutive model

Different from conventional VPSC method that simulating the macro stress–strain responses of pure metal or alloys, the proposed TMDT model only describes the plastic deformation behavior of a composite grain in MMCs. This composite grain includes reinforcement, metal matrix, and their interface, as well as the interface defects, including the defect heterogeneous distribution (gradient range), defect type (dislocations vs. twins), and defect volume fraction (twin spacing vs. twin volume). Since the spherical particle reinforcement is considered hard to deform, thermal mismatch stress at interface within a composite grain can be quantified. Thus, if combining with conventional VPSC method that aggregates all the plastic deformation behaviors of composite grains in MMCs, this TMDT model is not only suitable for understanding mechanical responses of MMCs, but also applicable to predict metallic materials with heterogeneous defect distribution within a grain.

### TMDT model description

Generally, the plastic deformation of a crystal with Face-Centered Cubic (FCC) crystal structure ascribes to its crystallographic slip. The plastic strain rate tensor for the crystal is related to the shear rate ${\dot{\gamma }}^{s}$ and the Schmid tensor ${m}_{ij}^{s}$ of the slip system *s*^37^:

1 $${\dot{\varepsilon }}_{ij} = {\sum }_{s}{\dot{\gamma }}^{s}{m}_{ij}^{s}=\dot{{\gamma }_{0}}{\sum }_{s}{m}_{ij}^{s}{\left(\frac{{m}_{pq}^{s}{\sigma }_{pq}}{{\tau }_{c}^{s}}\right)}^{n}sgn\left({m}_{pq}^{s}{\sigma }_{pq}\right),$$

where $\dot{{\gamma }_{0}}$ is a reference shear rate, *n* is the strain rate sensitivity,  ${\sigma }_{pq}$ is the stress tensor, and ${\tau }_{c}^{s}$ is the critical resolved shear stress (CRSS) of the slip system *s*. Different from conventional ${\tau }_{c}^{s}$ that is related to the initial dislocation density in metal, the ${\tau }_{c}^{s}$ in the current TMDT model describes the pre-existed thermal mismatch dislocation and twins in the composite grain of MMCs. Besides, the geometrical mismatch dislocation that induced by the geometrical mismatch between reinforcements and metal matrix is also considered. Moreover, the twins in the TMDT model are pre-implanted instead of generating during the deformation as that in traditional crystal plastic model. For conventional ${\tau }_{c}^{s}$, it is expressed as^17^:

2 $${\tau }_{c}^{s} = {\tau }_{0} + \chi \mu b\sqrt{{\rho }^{avg}},$$

where ${\tau }_{0}$ is the initial threshold stress, $\chi$ is the coefficient of dislocation interaction, $\mu$ is the shear modulus, $b$ is the Burgers vector of slip system *s*, and ${\rho }^{avg}$ is the initial average dislocation density in metal. Correspondingly, the ${\tau }_{c}^{s}$ in TMDT model is calculated by:

3 $${\tau }_{c}^{s} = {\tau }_{0} + {\tau }_{c}^{dis}+{\tau }_{c}^{twin}+ \chi \mu b\sqrt{{\rho }^{GM}},$$

where ${\tau }_{c}^{dis}$ and ${\tau }_{c}^{twin}$ are the contributions of thermal mismatch dislocation and twins, respectively, and ${\rho }^{GM}$ is density of geometrical mismatch dislocation. About ${\rho }^{GM}$, it can be presented by^7,19^:

4 $${\rho }^{GM} = 3\sqrt{\frac{5}{2}} {f}^\frac{1}{3}{\varepsilon }^{p}/(ab),$$

where $f$ is the reinforcement volume fraction, ${\varepsilon }^{p}$ is the plastic strain during deformation, and $a$ is the radius of the reinforcement. Based on Eqs. (1–4), it is clear that the mechanical behavior of the composite grain is mainly determined by the thermal mismatch dislocation and twins. We will then give a detailed description about these two factors.

**Figure 3:**
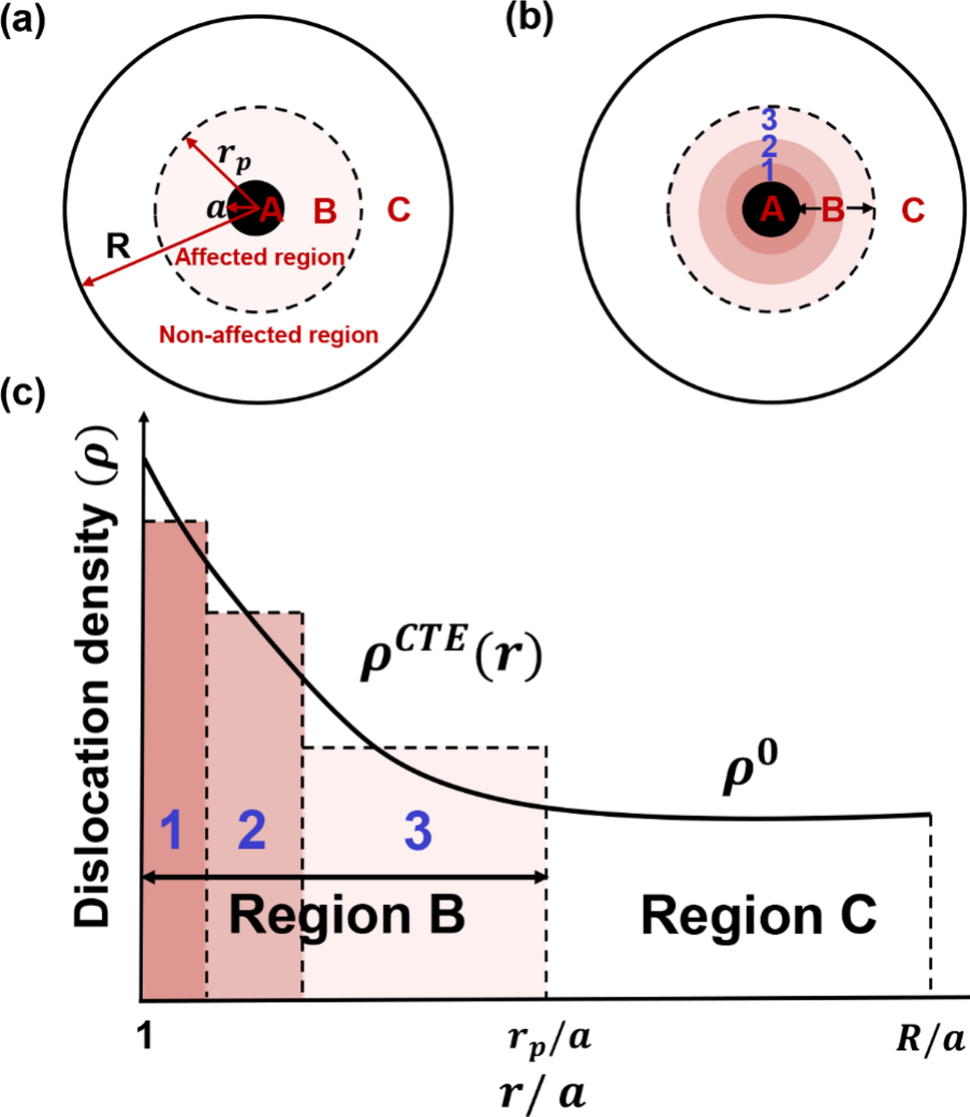
Schematic diagram of thermal mismatch induced dislocations in the TMDT model. (**a**) A representative grain with three regions: spherical particle reinforcement (region A), plastic accommodating region (region B), and non-affected region (region C). (**b**) Region B is further divided into three subregions considering gradient dislocation density distribution. (**c**) ${\rho }^{CTE} (r)$ in region B, showing dislocation density variation with the distance (*r*) that away from the interface, and ${\rho }^{0}$ is the initial average dislocation density in region C.

To better demonstrate the structure–property relationship of the composite grain in MMCs, we divide the composite grain in the TMDT model to three regions: spherical particle reinforcement (region A), plastic accommodating region (region B), and non-affected region (region C), as illustrated in Fig. 3a. In general, the stress that applied on the reinforcement during deformation can be calculated through the shear-lag model by considering the load transfer at the reinforcement/matrix interface^38,39^:

5 $${\sigma }_{ij}^{(A)} = \left(\frac{\omega }{2}f + 1\right){\sigma }_{ij}^{\left(C\right)},$$

where $\omega$ is the aspect ratio of the reinforcement, which equals to 1 when the reinforcement is spherical^40^. About $f$, it can be expressed by:

6 $$f= {\left(\frac{a}{R}\right)}^{3},$$

where $R$ is the radius of the grain.

About the thermal mismatch dislocation, ${r}_{p}$ in Fig. 3a indicates the range of dislocation distribution (region B) that can be further divided into finite subregions to manifest the heterogeneity of dislocation density. Figure 3b presents a showcase of three subregions in region B, and the variation of thermal mismatch induced dislocations is regarded as a function of the distance (*r*) that away from the reinforcement/matrix interface, and marked as ${\rho }^{CTE} (r)$ (Fig. 3c). ${\rho }^{0}$ is the initial average dislocation density in Region C. To numerically treat the heterogeneous dislocation distribution, the thermal mismatch affected region (region B) is divided to finite subregions. Of course, the more subregions are divided the more accurate the dislocation would be. We did try to use more subregions by sacrificing of computational efficiency of the model. However, the improvement on the modeling results is very limited (see Supplementary Fig. 1). Therefore, to balance both the accuracy and the computational efficiency, three subregions are selected in this work. The relationship between strain rate of each region (${\dot{\varepsilon }}_{ij}^{\left(k\right)}$) and the CRSS values (${\tau }_{c}^{(k)}$) can be established by Eq. (1). According to Eq. (3) and dislocation density hardening law, the ${\tau }_{c}^{(k)}$ is then expressed by^41^:

7 $${\tau }_{c}^{(k)} = {\tau }_{0} + {\tau }_{c}^{dis}+ \chi \mu b\sqrt{{\rho }^{GM}},$$

8 $${\tau }_{c}^{dis} = \chi \mu b\sqrt{{\rho }^{CTE}}.$$

The evolution of ${\rho }^{CTE}$ is closely related to the generation and annihilation process of dislocation, and can be expressed as^17^:

9 $$\frac{d{\rho }^{CTE}}{d\gamma } = {k}_{1}\sqrt{{\rho }^{CTE}}-{k}_{2}{\rho }^{CTE},$$

where ${k}_{1}$ is a rate-insensitive coefficient for dislocation generation, and ${k}_{2}$ is the rate sensitive coefficient for dynamic recovery. The relationship between ${k}_{1}$ and ${k}_{2}$ is described by^36^:

10 $$\frac{{k}_{2}\left(\dot{\varepsilon },T\right)}{{k}_{1}} = \frac{\chi b}{\mathrm{g}}\left(1 - \left(\frac{kT}{{\sigma }_{D}{b}^{3}}{\mathrm{ln}}\left(\frac{\dot{\varepsilon }}{\dot{{\varepsilon }_{0}}}\right)\right)\right),$$

where *k*, $\dot{{\varepsilon }_{0}}$, $\mathrm{g}$ and ${\sigma }_{D}$ are Boltzman's constant, a reference strain rate, a normalized effective activation enthalpy, and a drag stress, respectively.

**Figure 4:**
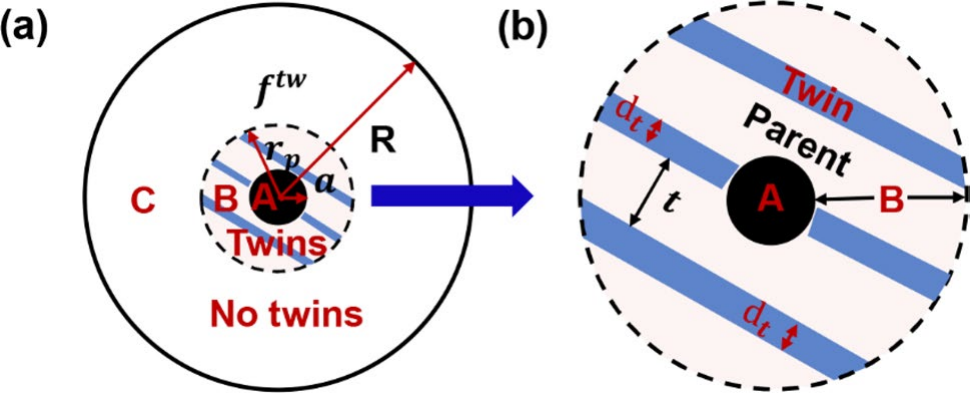
Schematic diagram of thermal mismatch induced twins in TMDT model. (**a**) A representative grain with three regions: spherical particle reinforcement (region A), plastic accommodating region (region B) and non-affected region (region C), where thermal mismatch induced nanoscale twins with volume fraction (${f}^{tw}$) existing in region B. (**b**) Enlarged region B shows average twin thickness (${d}_{t}$) and twin spacing (*t*), respectively.

Apart from the thermal mismatch dislocation, the thermal mismatch nanosclae twins are also considered in region B, as shown in Fig. 4. Different from deformation twins, the nanoscale twins here are treated as growth or annealing twins that are induced by thermal mismatch stress during the cooling process. Besides, the orientation of these twins is set exactly according to the twin-reorientation relative to the parent grain. The crystallographic orientation relationship between the parent grain and the thermal mismatch twins, and the shape and volume fraction (${f}^{tw}$) of the nanoscale twins remain unchanged in the simulated deformation process. Regarding the twin related specific parameters, the thickness and average spacing of the twins are indicated by ${d}_{t}$ and *t*, respectively. Similarly, the strain rate of the parent grain (${\dot{\varepsilon }}_{ij}^{parent}$) and twins (${\dot{\varepsilon }}_{ij}^{twin}$) can be deduced according to the CRSS of the same activated slip systems in Eq. (1). For the twin case in Fig. 4, the barrier effects of nanoscale twins to the dislocation motion is generally influenced by ${\tau }_{c}^{(k)}$ and the twin characteristics^42^:

11 $${\tau }_{c}^{(k)} = {\tau }_{0} +{\tau }_{c}^{twin}+ \chi \mu b\sqrt{{\rho }^{GM}},$$

12 $${\tau }_{c}^{twin} =\frac{K}{\sqrt{{d}_{mfp}}}= \mu {HP}^{\alpha }\sqrt{\frac{b}{{d}_{mfp}}},$$

where *K* is the Hall–Petch coefficient. ${HP}^{\alpha }$ is a dimensionless constant indicating $\alpha$ type of slip dislocations that impinge on grain boundaries, and ${d}_{mfp}$ is the mean free path of the slip system. When there are no twins in grain, ${d}_{mfp}$ equals to the grain size, but when there are twins in grain, ${d}_{mfp}$ equals to the twin spacing ($t$). In addition, since the parent orientation may be influenced by the twins, the effect of parent texture is also considered by controlling the Euler angle of each grain in the TMDT model.

Right now, each composite grain in MMCs with heterogeneous thermal mismatch dislocation and twins can be well described through the TMDT model. Thus, the average Cauchy stress of the composite grain (Figs. 3 and 4), ${\sigma }_{ij}^{g}$, is expressed:

13 $${\sigma }_{ij}^{g} = {\sum }_{k=A,B1-3,C}{f}^{\left(k\right)}{\sigma }_{ij}^{(k)},$$

where ${f}^{\left(k\right)}$ and ${\sigma }_{ij}^{(k)}$ are the volume fraction and Cauchy stress of region *k* (*k*: A, B1–3, C), respectively. And when region B containing thermal mismatch twins, its Cauchy stress is calculated through^42,43^:

14 $${\dot{\varepsilon }}_{ij}^{\left(B\right)} = \left(1 - {f}^{tw}\right){\dot{\varepsilon }}_{ij}^{parent} + {f}^{tw}{\dot{\varepsilon }}_{ij}^{twin},$$

15 $${f}^{tw} = \frac{2{d}_{t}}{t+2{d}_{t}}.$$

By combining the VPSC method, each region in the composite grain is regarded as a visco-plastic inclusion that embedded in a homogeneous equivalent medium with effective visco-plastic behavior, and this medium represents the aggregate of all the composite grains in MMCs. Therefore, the strain rates (${\dot{\varepsilon }}_{ij}^{(k)}$) and stresses of single region are related self-consistently to the corresponding values of the medium as follows:

16 $${\dot{\varepsilon }}_{ij}^{(k)}-{\overline{\dot{\varepsilon }} }_{ij}=-{\tilde{M }}_{ijpq}\left({\sigma }_{pq}^{\left(k\right)}-{\overline{\sigma }}_{pq}\right),$$

where ${\overline{\dot{\varepsilon }} }_{ij}$ and ${\overline{\sigma }}_{pq}$ are the macroscopic strain rate and stress of the medium, respectively, ${\tilde{M }}_{ijpq}$ is interaction tensor between the region and medium, can be expressed by:

17 $${\tilde{M }}_{ijpq}={\left(I-{S}_{ijpq}\right)}^{-1}{S}_{ijpq}{\overline{M} }_{ijpq}.$$

Here, *I* is the identity tensor, ${S}_{ijpq}$ is the visco-plastic Eshelby tensor for a given region, and $\overline{M }$ is the visco-plastic compliance for the medium. And the self-consistent method assumes the behavior of the medium has a linear relation analogical to:

18 $${\overline{\dot{\varepsilon }} }_{ij}=-{\overline{M} }_{ijpq}{\overline{\sigma }}_{pq}+{\dot{\varepsilon }}_{ij}^{0},$$

where ${\dot{\varepsilon }}_{ij}^{0}$ is the back extrapolated term. Finally, by combining with the Eqs. (1, 5–10, 13, 16–18)and Eqs. (1, 5, 6, 11–18), the separate effects of thermal mismatch dislocation and twins on mechanical properties of MMCs can be estimated based on the VPSC-TMDT model.

### Comparison of influential factors in VPSC-TMDT model, FEM and VPSC methods

**Figure 5:**
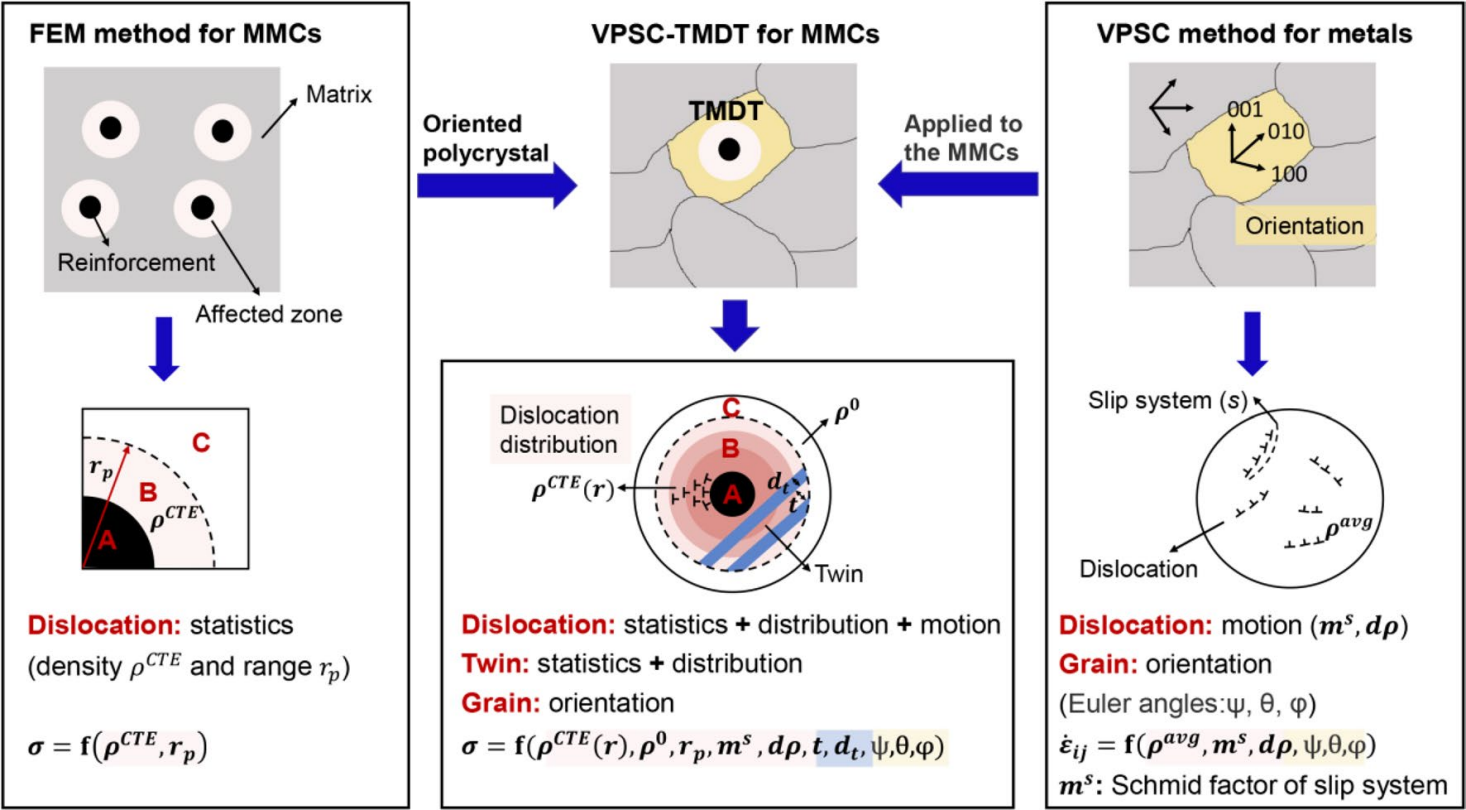
Schematics showing the differences of influential factors in VPSC-TMDT model, FEM, and VPSC methods. About the element in the TMDT model, it combines the spatial structure in that of FEM method and the physical mechanism of VPSC method, with emphasizing on the heterogeneous dislocation distribution and nanoscale twins in the thermal mismatch affected region.

Figure 5 shows differences between the influential factors in VPSC-TMDT model, FEM, and VPSC methods. The detailed descriptions of FEM and VPSC methods to calculate the mechanical stress–strain responses of MMCs are stated in Supplementary Note 1 and 2, respectively. For the FEM method, it mainly considers the dislocation distribution range and the statistical dislocation density. However, grain characteristics such as the heterogeneous distribution of the dislocations are not considered. In comparison, the element in VPSC method considers the behaviors of dislocations, twins, and their interactions in the polycrystal metals. In addition, both the shape and orientation of the grains and twins are also taken into account. Nevertheless, individual grains with spherical elastic particle and associated plastic heterogeneity is not considered. Therefore, the element in VPSC-TMDT model combines the spatial structure in that of FEM method and the physical mechanism in VPSC method, with special emphasizing in the thermal mismatch affected region. And for MMCs, their macro mechanical stress–strain responses may be simulated and predicted when simultaneously considering the thermal mismatch induced dislocations and twins.

## Results and discussions

In order to demonstrate the validity and capability of the VPSC-TMDT model, the most studied SiC/Al composites with thermal mismatch dislocations or twins are simulated and compared with the experimental results. Besides, the effects of heterogeneous dislocation distribution and nanoscale twin characteristics on mechanical properties of the SiC/Al composites are compared in details, with basic slip systems ({111} < 110 >) of FCC crystal.

### VPSC-TMDT model with dislocations

Generally, SiC/Al composites with the micro-sized and low volume fraction of SiC particles, dislocations in the thermal mismatch affected region are usually generated (as shown in Fig. 1). Here, the 15 vol.% SiC/A356 composites with the SiC size of 7.5 and 16 μm^44^ are chosen to verify the VPSC-TMDT model that considering thermal mismatch dislocations.

**Figure 6:**
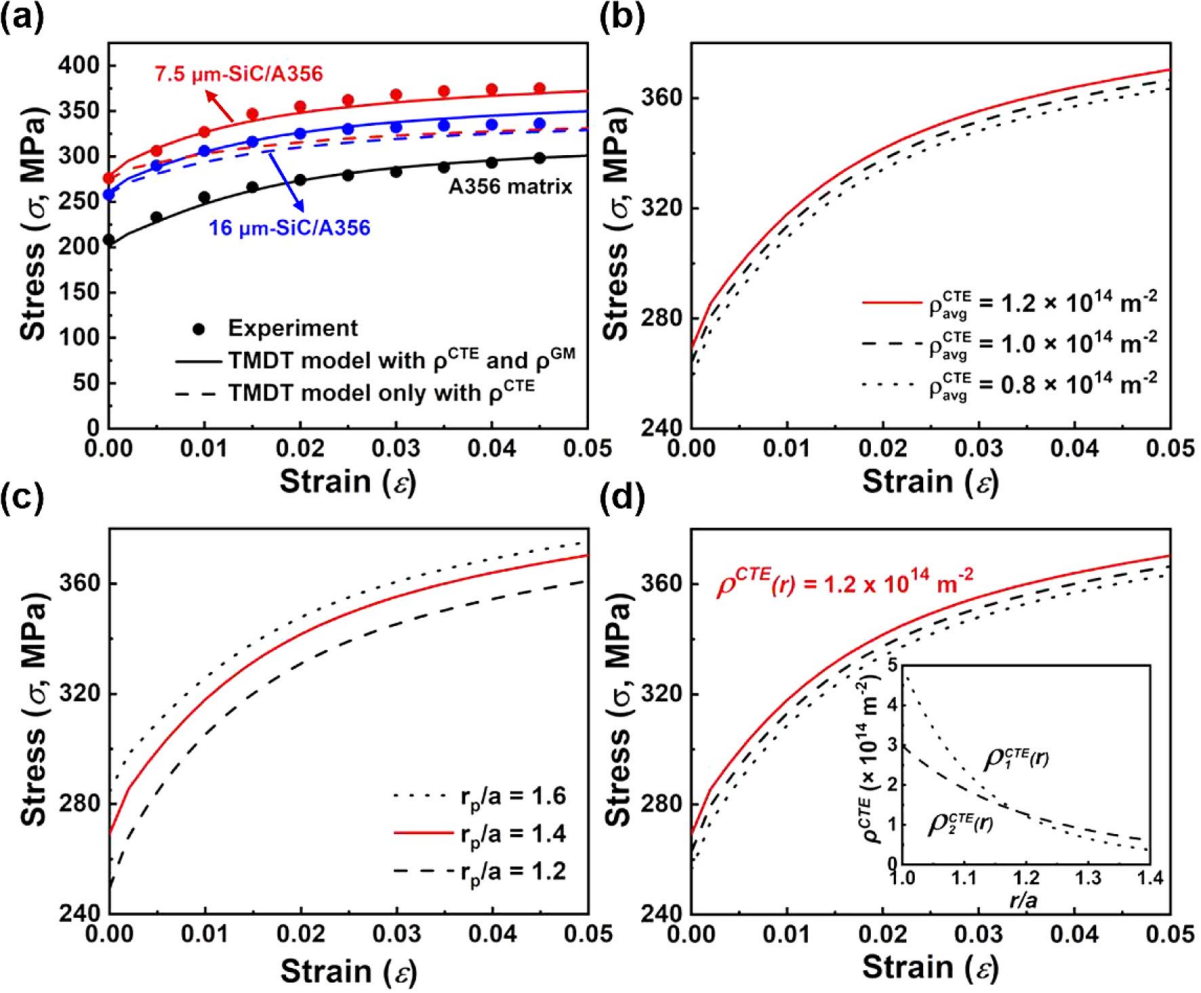
The mechanical stress–strain responses calculated by the VPSC-TMDT model with thermal mismatch dislocations. (**a**) The calculated results by the VPSC-TMDT model well correspond to the experimental data of the SiC/A356 composites. (**b**) Mechanical responses with various average dislocation density (${\rho }_{avg}^{CTE}$). (**c**) Mechanical responses with various ranges of dislocation distributions. (**d**) Mechanical responses with various heterogeneous dislocation distributions.

Prior to consider composites, the mechanical strength simulated by the VPSC-TMDT model is first compared with the experimental tensile stress–strain responses of the A356 matrix, as shown in Fig. 6a. The dislocation density hardening related parameters are estimated and summarized in Table 1. Meanwhile, ${\rho }_{avg}^{CTE}$ and ${r}_{p}$ are calculated according to the spherical dislocation punching model^45^ (see Supplementary Table 1 and Supplementary Note 3 for details). Then, by considering parameters in Table 1 and the calculated ${\rho }_{avg}^{CTE}$ and ${r}_{p}$, the mechanical responses of the SiC/A356 composites are plotted. Figure 6a shows that a significant deviation exists when the geometry mismatch dislocations are not included in the VPSC-TMDT model (as shown by the blue and red dash lines). Based on Eq. (9), the annihilation rate of dislocation will eventually comparable with the generation rate, and thus a steady-state value of ${\rho }_{\alpha }^{CTE}$ is reached, where $\mathrm{\alpha }$ means a particular slip mode of the crystal^42^. Before this steady-state plastic deformation stage, the value of ${\rho }^{CTE}$ will continue increasing with the increased strain, but exhibits a continuously decreased tendency after this stage^46^. Based on the variation of ${\rho }^{CTE}$ in the TMDT model (Supplementary Fig. S2), it can be inferred that when $\upvarepsilon$ reaches 2.5% and 4.0%, SiC/A356 composites (7.5 μm and 16 μm particle size) without ${\rho }^{GM}$ remain in the stage of stable plastic deformation. However, since the corresponding experimental results inevitably involve plastic deformation induced geometry mismatch dislocations, the simulated results well correspond with the experimental results after addition of the parameters that contributed by the geometry mismatch dislocations. This indicates the enhanced accuracy of the TMDT model when simultaneously considering the thermal mismatch and geometry mismatch dislocations. Note that, in the VPSC-TMDT model, it considers the perfect interface bonding state of MMCs, if there exist some geometrical defects at the interface, such as micro-cracks and voids, the VPSC-TMDT model probably may not be suitable to calculate the mechanical properties of MMCs.

**Table 1:** The dislocation density hardening parameters of the A356 matrix and the 15 vol.% SiC/A356 composites.

Materials	${\uptau }_{0} \;(\mathrm{MPa})$	${\uprho }^{0} \;({\mathrm{m}}^{-2})$	${\mathrm{k}}_{1} \; (\mathrm{m})$	$\mathrm{g}$	${\upsigma }_{\mathrm{D}} \; (\mathrm{MPa})$
A356	66	5.e11	2.e9	1.5e−2	300
SiC/A356 (7.5 μm)	66	5.e12	2.e9	1.5e−2	300
SiC/A356 (16 μm)	66	5.e12	2.e9	1.5e−2	300

As shown in Fig. 6b–d, the effect of ${\rho }_{avg}^{CTE}$ and ${r}_{p}$, as well as heterogeneous dislocation distribution (region B) on mechanical responses of 7.5 μm SiC/A356 composites are studied. Figure 6b shows that with the same ${r}_{p}$, the mechanical strength of the composites increases with the increment of ${\rho }_{avg}^{CTE}$. In comparison, when fixing the same ${\rho }_{avg}^{CTE}$, the mechanical strength is also found presenting a corresponding trend with the increased ${r}_{p}$ (Fig. 6c). Both these trends are consistent with the results that simulated by the existed FEM models^19^, indicating the suitability of the proposed VPSC-TMDT model. Finally, we focus on the effect of heterogeneous dislocation distribution. To demonstrate the influence of this parameter, two different density distributions, ${\rho }_{1}^{CTE}(r)$ and ${\rho }_{2}^{CTE}(r)$, are used for comparison. The ${\rho }_{avg}^{CTE}$ values of these two distributions are both equal to 1.2 × 10^14^ m^−2^. As shown in the inset chart of Fig. 6d, although the decreasing rate of ${\rho }_{1}^{CTE}(r)$ is higher than that of ${\rho }_{2}^{CTE}(r)$, the mechanical strength of the composites with ${\rho }_{2}^{CTE}(r)$ distribution is superior to that with ${\rho }_{1}^{CTE}(r)$ distribution. This phenomenon can be understood based on the difference of CRSS between these two distributions. The value of ${\tau }_{c}^{(B)}$ is calculated by the average value of CRSS in region B1–3, and ${\tau }_{c}^{(B)}=\left({f}^{(B1)}{\tau }_{c}^{(B1)}+{f}^{(B2)}{\tau }_{c}^{(B2)}+ {f}^{(B3)}{\tau }_{c}^{(B3)}\right)/{f}^{(B)}$. Based on Eqs. (7, 8), the CRSS values in region B1–3 are determined by the square root of dislocation density ($\sqrt{{\rho }^{CTE}}$). Thus, ${\tau }_{c}^{(B)}$ can be calculated through ${\tau }_{c}^{(B)}={\tau }_{0} + \chi \mu b\sqrt{{\rho }^{GM}}+\chi \mu b \left({f}^{(B1)}\sqrt{{\rho }_{B1}^{CTE}}+ {f}^{(B2)}\sqrt{{\rho }_{B2}^{CTE}}+ {f}^{(B3)}\sqrt{{\rho }_{B3}^{CTE}}\right)/{f}^{(B)}$. Hence, the lower decreasing rate of ${\rho }^{CTE} (r)$ can lead to a higher value of $\left({f}^{(B1)}\sqrt{{\rho }_{B1}^{CTE}}+ {f}^{(B2)}\sqrt{{\rho }_{B2}^{CTE}}+ {f}^{(B3)}\sqrt{{\rho }_{B3}^{CTE}}\right)/{f}^{(B)}$, namely enhancing the value of ${\tau }_{c}^{(B)}$ (higher mechanical strength). Meanwhile, when further reducing the decreasing rate of ${\rho }^{CTE} (r)$ to zero, which means a completely uniform dislocation distribution, the calculated mechanical strength of the composites is the highest (as makes by the red line in Fig. 6d). Therefore, based on the calculated results, homogeneous dislocation distribution may provide the upper-bond of mechanical strength compared to that of  heterogeneous dislocation distribution.

### VPSC-TMDT model with twins

**Figure 7:**
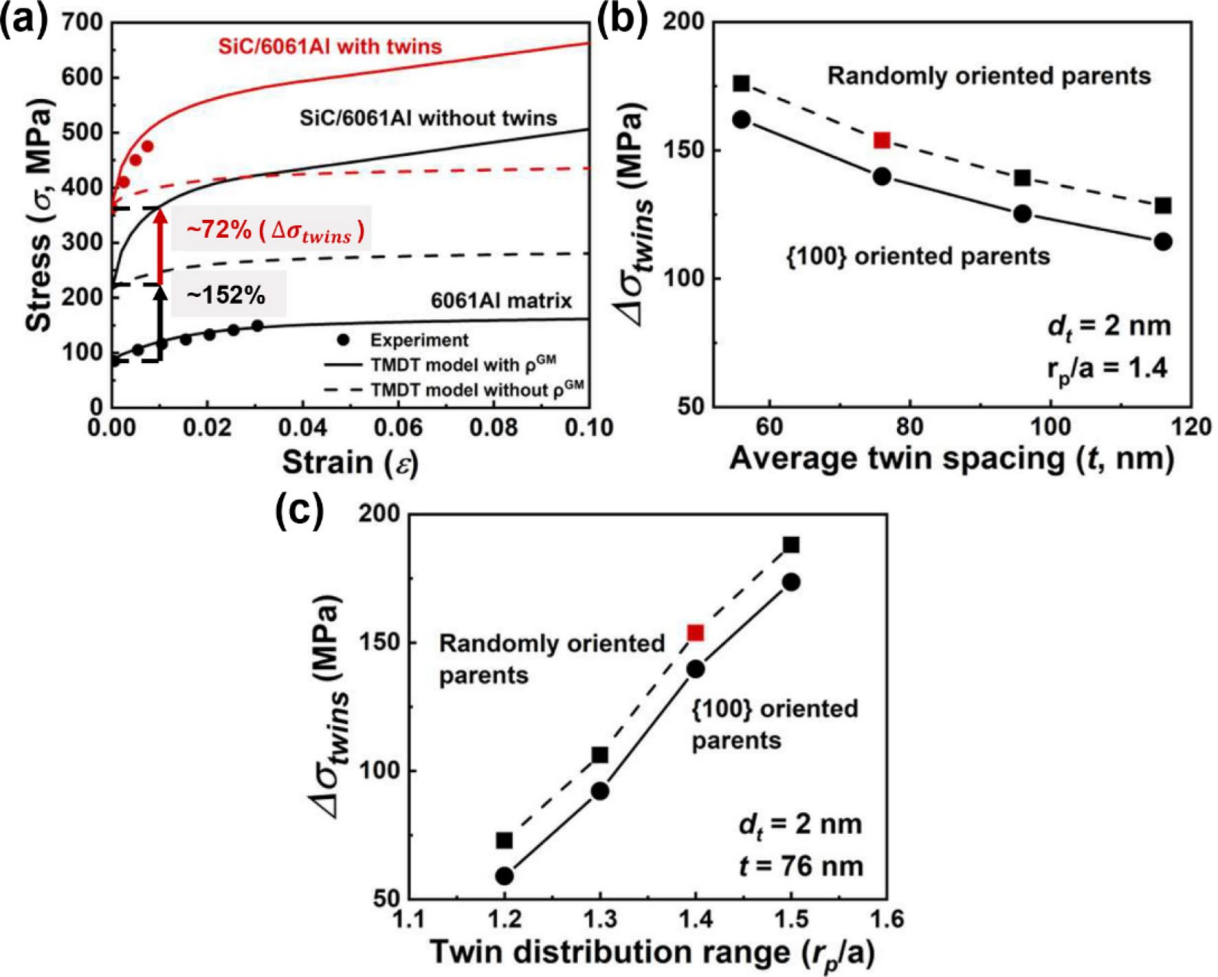
The mechanical stress–strain responses calculated by the VPSC-TMDT model with thermal mismatch twins. (**a**) Compared to the SiC/6061Al composites with and without twins, the mechanical properties of the composites after embedding twins are significantly enhanced. (**b**,**c**) Mechanical strength of the composites is less influenced by parent texture, but strong dependent on the twin spacing (*t*) and twin distribution range (${r}_{p}/a$).

In order to demonstrate the validity of the VPSC-TMDT model on thermal mismatch nanoscale twins, the 30 vol.% SiC/6061Al composites with average SiC particle size of 150 nm are studied^8^ (Fig. 1). Similar to the aforementioned dislocation study, the values of the dislocation density hardening parameters of the 6061Al matrix are first estimated and summarized in Table 2 based on the published experimental studies. Besides, the {111} twin characteristics of the composites are also considered based on experimental transmission electron microscopy (TEM) characterization results (see Supplementary Fig. S3 for details). Then, by considering parameters in Table 2 and twin characteristics (${d}_{t}$ and *t*), mechanical responses of the SiC/6061Al composites are plotted by the VPSC-TMDT model, as shown in Fig. 7. Notably, due to the parent orientation may be influenced by the twins^42^, the effect of parent texture on the mechanical strength of the composites is therefore also considered. Figure 7a illustrates that the ${\sigma }_{y}$ values of the SiC/6061Al composites are much higher when compared to the 6061Al matrix. For the composites without twins, the ${\sigma }_{y}$ value increases from 85 to 215 MPa (~ 152% enhancement) due to the fine grain strengthening and load transfer.  After embedding the {111} orientated twins, the value of ${\sigma }_{y}$ further increases to 369 MPa, displaying a significant twin strengthening phenomenon. Similar to the results in Fig. 6a, the accuracy of the simulated results is also improved after adding the geometry mismatch dislocations contributed strength, as evidenced by the decreased deviation in Fig. 7a  (the red and black dash lines mean the mechanical properties without addition of geometry mismatch dislocations).

**Table 2:** The dislocation density hardening parameters of the 6061Al matrix and the 30 vol.% SiC/6061Al composites.

Materials	${\uptau }_{0} \; (\mathrm{MPa})$	${\uprho }^{0} \; ({\mathrm{m}}^{-2})$	${\mathrm{k}}_{1} \; (\mathrm{m})$	$\mathrm{g}$	${\upsigma }_{\mathrm{D}} \; (\mathrm{MPa})$
6061Al	27	5.e11	1.5e9	1.5e−2	200
SiC/6061Al	27	5.e12	1.5e9	1.5e−2	200

Figure 7b shows that when decreasing *t* of the twins under the condition of fixed ${d}_{t}$ and twin distribution range (also means the increased twin volume fraction, ${f}^{tw}$), the simulated strength exhibits an increasing trend, which ascribes to that the dislocation movement can be blocked by the increased number of twin boundaries. Besides, we also consider the effect of parent texture on the mechanical strength, and the randomly oriented and {100} oriented parents are set for comparison. The results imply that the mechanical strength is almost independent on the parent texture. Analogous to the strength variation with the distribution range of dislocations (Fig. 6c), the strength of the composites is simulated likewise increasing with the increased twin distribution range (Fig. 7c). Therefore, it may conclude that whether for the weak or strong textured parent in the grain, embedding twins and meanwhile optimizing their specific parameters could remarkably improve the mechanical strength of MMCs.

Apart from the calculation through VPSC-TMDT model, the mechanical strength of SiC/Al composites is also calculated and compared by the conventional FEM and VPSC methods (as shown in Supplementary Fig. S4). It is found that, when considering the thermal mismatch dislocations, the calculation results of FEM method and VPSC-TMDT model almost keep the same trend. Nevertheless, compared to the FEM method, the VPSC-TMDT model still can study the effects of heterogeneous dislocation distribution and thermal mismatch twins on mechanical properties of MMCs (Figs. 6d and 7). On the other hand, for the conventional VPSC method, it can only calculate the strength of the A356 matrix, since the thermal mismatch defects are not considered in its element. Therefore, the proposed element in VPSC-TMDT model combines the spatial structure in that of FEM method and the physical mechanism in VPSC method.

### Mechanical properties prediction of the Gr/Cu composites

Due to the intrinsic characteristics of high strength and low CTE, as well as excellent functional properties, carbon nanomaterials, such as carbon nanotubes (CNTs), Gr, and carbon nanofibers, have been increasingly adopted for preparing the reinforced nanocomposites^47,48^. In addition, compared to the SiC particles, the nanocarbon materials own much higher modulus, and therefore producing larger interfacial thermal mismatch stress at the nanocarbon/metal interfaces. One can image that even if under the condition of much smaller volume fraction, the unique features of the nanocarbon materials may introduce nanotwins in the thermal mismatch affected region of the composites. Here, to confirm this point, the non-reactive system of Gr/Cu composites is fabricated (see Supplementary Note 4 for details), and the microstructures and mechanical properties of the as-fabricated composites are also examined and predicted through the VPSC-TMDT model.

**Figure 8:**
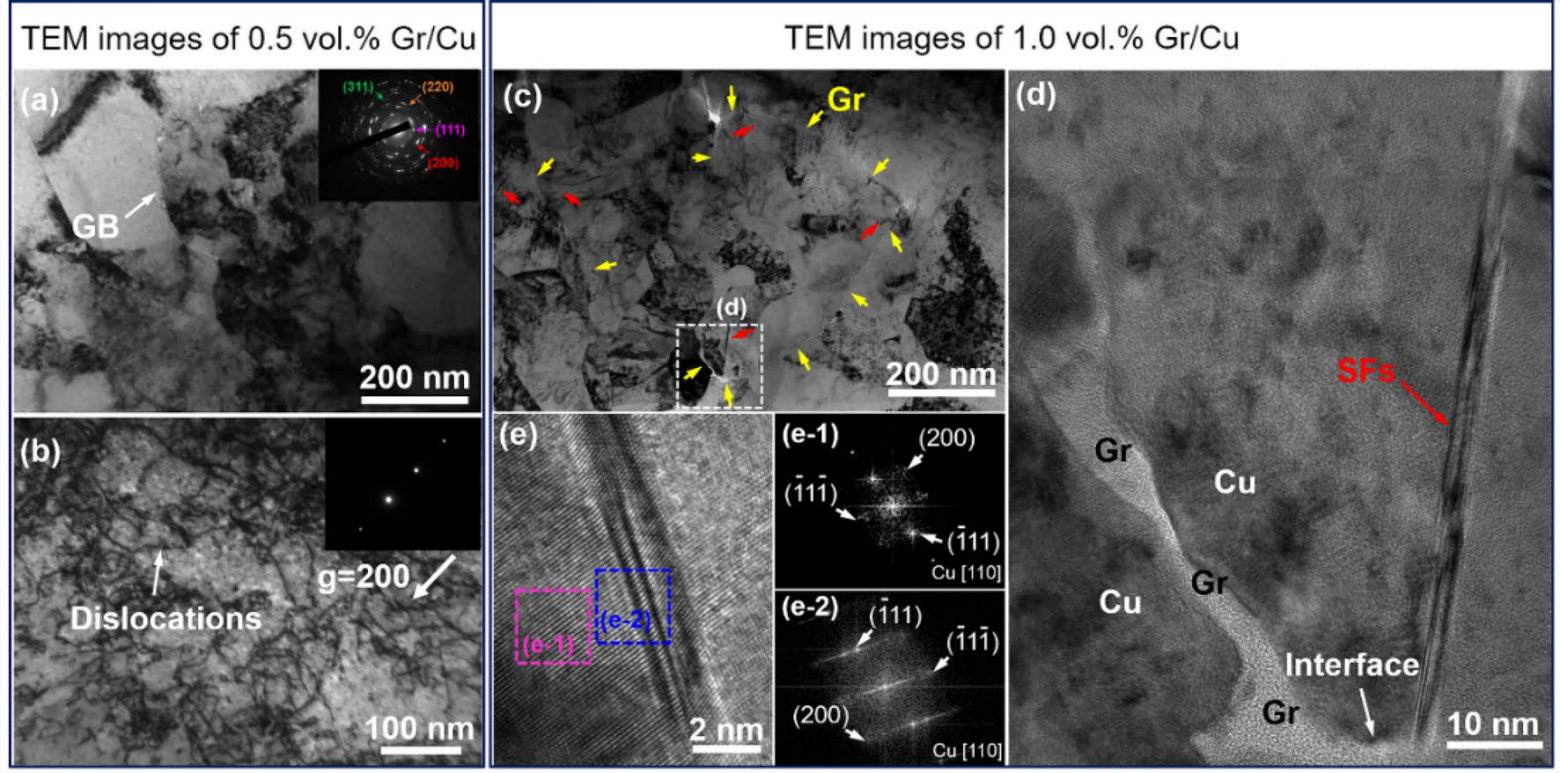
The TEM images of the Gr/Cu composites with different contents of Gr. (**a**,**b**) For the 0.5 vol.% Gr/Cu composites, the thermal mismatch dislocations are observed under two-beam condition with the **g** vector (200) of Cu. (**c**–**e**) For the 1.0 vol.% Gr/Cu composites, the interfacial thermal mismatch defect type transforms from dislocations to nanotwins, which are identified by the SAED patterns in e-1 and e-2.

Figure 8 shows the TEM images of the Gr/Cu composites. According to the SEM results in Supplementary Fig. S5, the Gr is uniformly distributed within the composites. For the 0.5 vol.% Gr/Cu composites in Fig. 8a,b, the thermal mismatch dislocations are clearly observed under two-beam condition with the **g** vector (200) of Cu. The inset selected area electron diffraction (SAED) pattern in Fig. 8a confirms the existence of nanocrystalline Cu grains, which may form due to the recrystallization of the Cu matrix. Differently, when the content of Gr is increased to 1.0 vol.%, the dislocations are hard to see. Instead, around the Gr/Cu interfaces (as marked by the yellow arrows in Fig. 8c), there exist a large density of black “lines” (indicated by the red arrows in Fig. 8c). The magnified TEM image in Fig. 8d shows that the black “line” starts from the Gr/Cu interface and extends into the Cu grain interior, displaying a typical characteristic of epitaxial growth. Figure 8e gives the high-resolution TEM (HRTEM) image of the black “line”, and the corresponding SAED patterns in Fig. 8e-1 and e-2 reveal that the black “line” is nanotwins [also nano stacking faults (SFs)] in nature. That is to say, when the volume fraction of Gr increases from 0.5 to 1.0 vol.%, the thermal mismatch defect type transforms from dislocations to twins. Compared to the content of SiC particle in Ref.^8^, the Gr content is far smaller. Also, further experimental and theoretical studies are needed to reveal the underlying mechanism for the generation of nanotwins.

**Figure 9:**
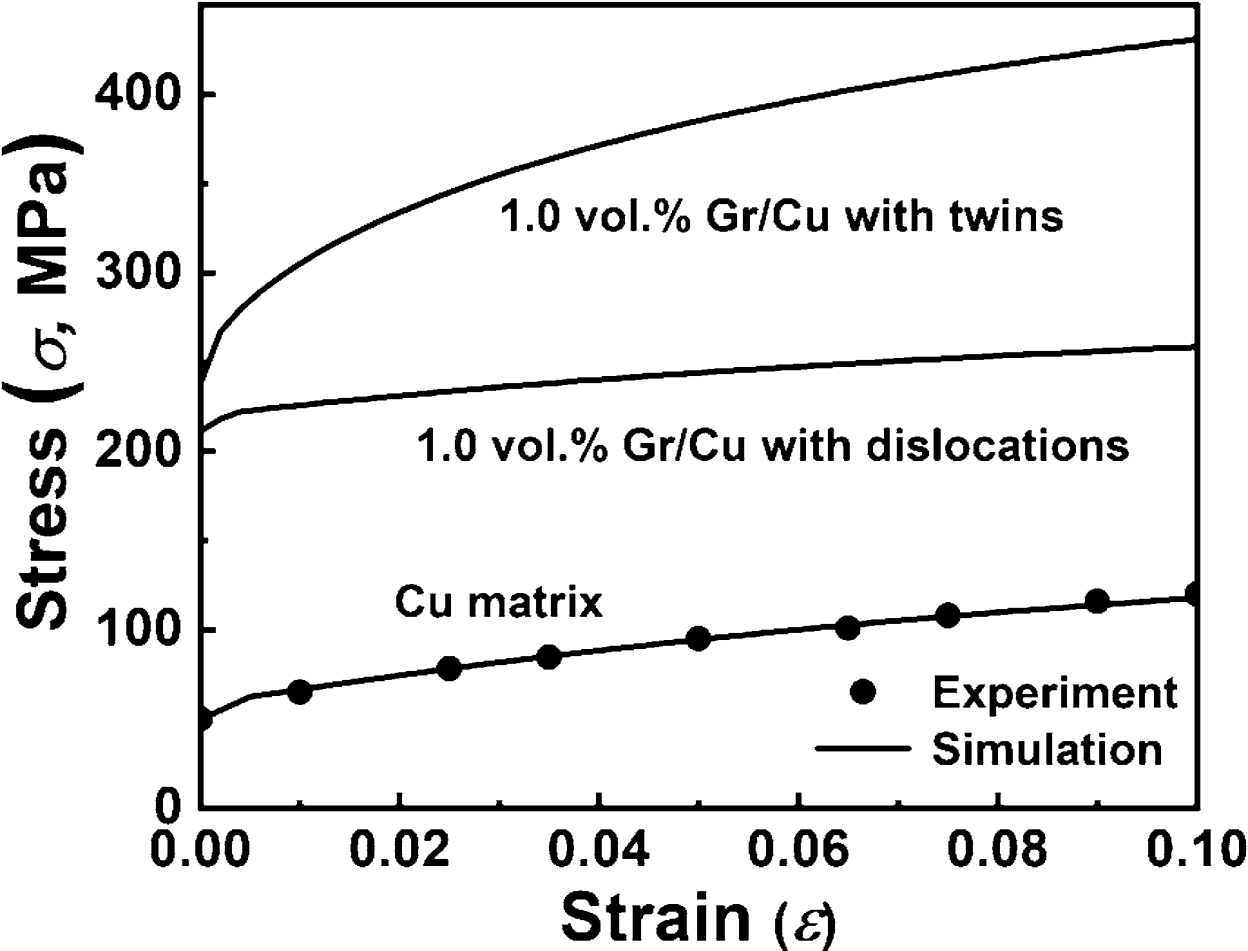
The mechanical properties of the Gr/Cu composites simulated by the VPSC-TMDT model. The results show that under the same conditions, the strengthening effect of the thermal mismatch twins is superior to that of dislocations.

To further evaluate the effect of defect type on mechanical properties of the Gr/Cu composites, the VPSC-TMDT model is employed. Notably, in the VPSC-TMDT simulation, the Gr is treated as a particle with the radius of ~ 50 nm according to the Gr length in the TEM result of Fig. 8d. As shown in Fig. 9, the mechanical property of the pure Cu is first fitted based on the experimental result in Ref.^49^, and the obtained dislocation density hardening parameters are given in Table 3. Meanwhile, by inputting the calculated dislocation and twin related specific parameters (Supplementary Table 2 and Supplementary Note 3) into the VPSC-TMDT model, the mechanical properties of the 1.0 vol.% Gr/Cu composites are predicted. Obviously, relative to the Cu matrix, the Gr/Cu composites exhibit much higher strength. Besides, the ${\sigma }_{y}$ of the 1.0 vol.% Gr/Cu composites that with thermal mismatch twins is 237 MPa, which is ~ 12.3% higher than that with dislocations. Through this direct comparison, it may infer that under the same condition, the thermal mismatch twins may be more effective than the dislocations in strengthening mechanical properties of MMCs.

**Table 3:** The dislocation density hardening parameters of the Cu matrix and the 1 vol.% Gr/Cu composites.

Materials	${\uptau }_{0} \; (\mathrm{MPa})$	${\uprho }^{0} \; ({\mathrm{m}}^{-2})$	${\mathrm{k}}_{1} \; (\mathrm{m})$	$\mathrm{g}$	${\upsigma }_{\mathrm{D}} \; (\mathrm{MPa})$
Cu	13	5.e11	0.2e9	1.5e−2	200
Gr/Cu	13	5.e12	0.2e9	1.5e−2	200

Actually, as a kind of structure and function integration material, the thermal and electrical conductivities of the Gr/Cu composites have also attracted extensive attention in the past few years^50,51^. The results show that although the dislocations are usually distributed near the Gr/Cu interfaces, the enhancement of thermal conductivity of the composites is still up to ~ 22% compared to the Cu counterpart^50^. It is universally known that relative to the dislocations, the scattering strength of the twin boundaries to the electrons and phonons are far lower. Therefore, if substituting twins for dislocations in the thermal affected region of MMCs, it is expected to prepare such a nanocomposite that is endowed with both ultrahigh strength and ultrahigh thermal and electrical conductivities.

## Conclusions

In this work, we propose a physical-based crystal plasticity model that simultaneously considering the thermal mismatch dislocations and twins (TMDT). To verify the validity and capability of the VPSC-TMDT model in predicting the mechanical properties of metal matrix composites (MMCs), the SiC/A356 composites with thermal mismatch dislocations and SiC/6061Al composites with thermal mismatch twins are studied. The results show that the simulation results of VPSC-TMDT model can match well with the experimental data. For the SiC/A356 composites, increasing the density and distribution range of the dislocations can enhance both the strength of the composites, which is consistent with the results in the existed FEM methods. Besides, the VPSC-TMDT also suggests that the uniform distribution of dislocations is more conducive to the mechanical property improvement. For the SiC/6061Al composites that with thermal mismatch twins, it is uncovered that their strength increases with the increased twin volume fraction and distribution range, but is independent on the parent texture. The VPSC-TMDT is also applied to predict the strength of Gr/Cu composites and the results display that, for the 1.0 vol.% Gr/Cu composites, the yield strength can be enhanced ~ 12.3% when the thermal mismatch dislocations are fully transformed to twins. The present findings may provide an alternative aim to the future preparation of MMCs, that is, substituting twins for dislocations in the thermal mismatched affected region. Also, this approach is closely related to optimizations of the reinforcements and fabrication techniques.

## Supplementary Information

Supplementary Information.
