# Supplementary material for: A crystal plasticity model for metal matrix composites considering thermal mismatch stress induced dislocations and twins
Source: Sci Rep. 2021 Aug 6;11:16053. doi: 10.1038/s41598-021-95439-z (PMC8346628; doi:10.1038/s41598-021-95439-z)
Supplement: Supplementary file 1 — Supplementary Information. [file 41598_2021_95439_MOESM1_ESM.docx]

**Supplementary Information for**

**A crystal plasticity model for metal matrix composites considering thermal mismatch stress induced dislocations and twins**

Y.N. Hou^1*^, K.M. Yang^1*^, J. Song^1^, H. Wang^2^, Y. Liu^1^, T.X. Fan^1^

1 State Key Laboratory of Metal Matrix Composites, School of Materials Science and Engineering, Shanghai Jiao Tong University, Shanghai 200240, China

2 State Key Laboratory of Mechanical System and Vibration, Shanghai Jiao Tong University, Shanghai 200240, China

*These authors contribute equally to this work

Corresponding authors: [wanghm02@sjtu.edu.cn](mailto:wanghm02@sjtu.edu.cn), [yliu23@sjtu.edu.cn](mailto:yliu23@sjtu.edu.cn), [txfan@sjtu.edu.cn](mailto:txfan@sjtu.edu.cn)


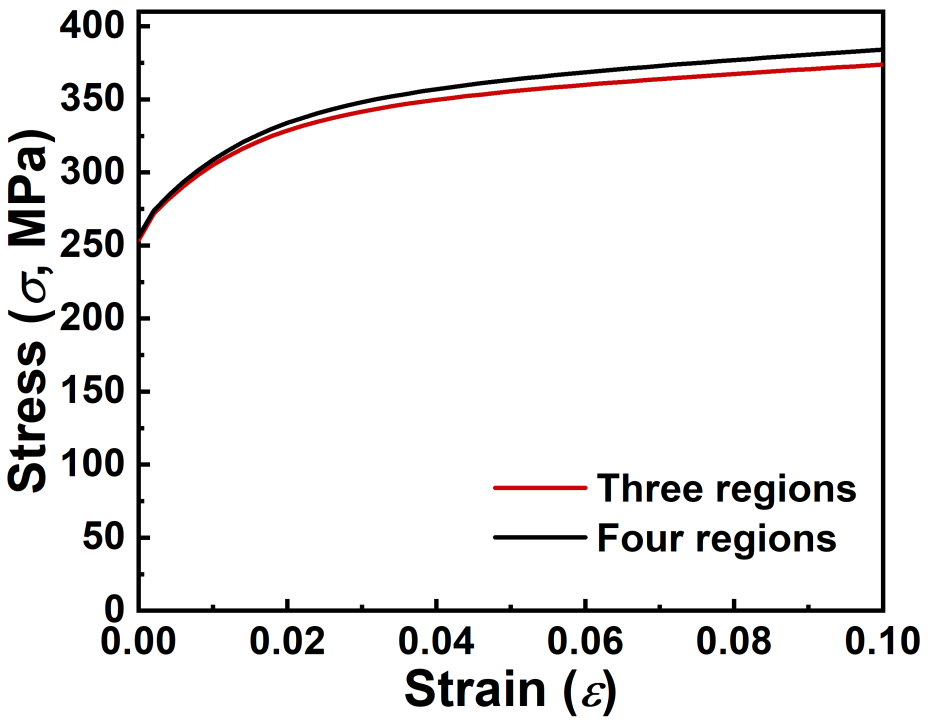


**Supplementary Figure 1.** The influence of three and four subregions in region B on mechanical properties of SiC/Al composites.


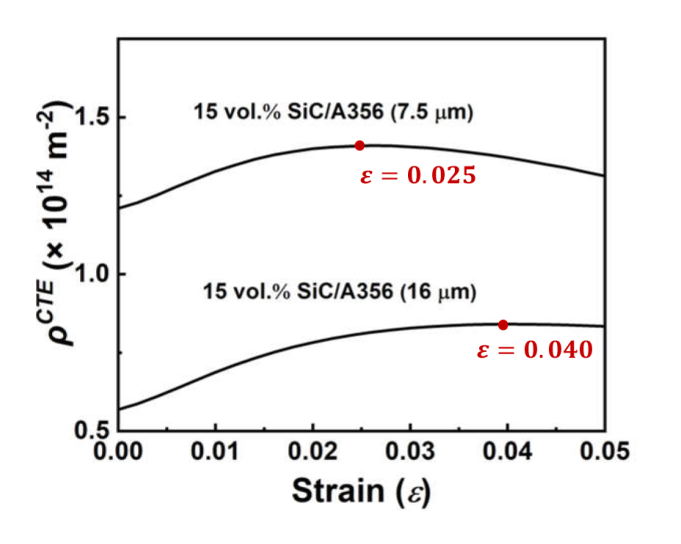


**Supplementary Figure 2. The variation of** $\boldsymbol{\rho}^{\boldsymbol{CTE}}$ **with the tensile strain (**$\boldsymbol{\varepsilon}$**).** For the 15 vol.% SiC/A356 composites with 7.5 μm SiC, a steady-state appears when $\varepsilon$ is 0.025, but in the situation of 16 μm SiC, the critical $\varepsilon$ increases to 0.04. At the steady-state, the composites will enter a stage of stable plastic deformation.


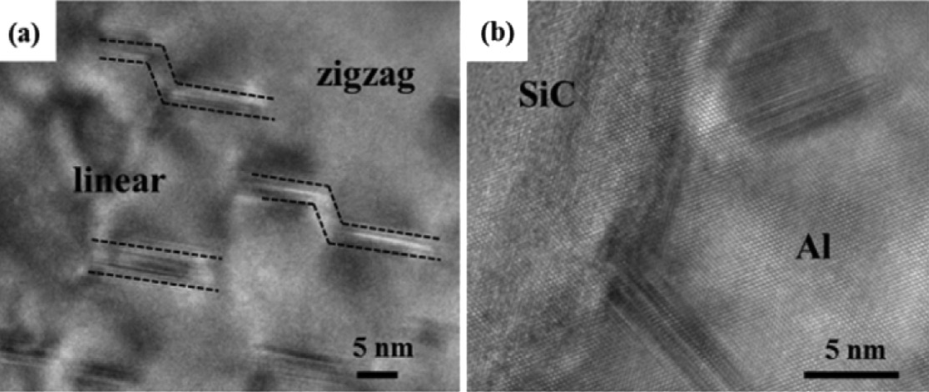


**Supplementary Figure 3. The HRTEM images of the 30 vol.% SiC/6061Al composites.** The results showing that (a) the shape of the produced nanotwins is linear and zigzag and (b) the average thickness of the nanotwins ($d_{t}$) is ~2 nm.


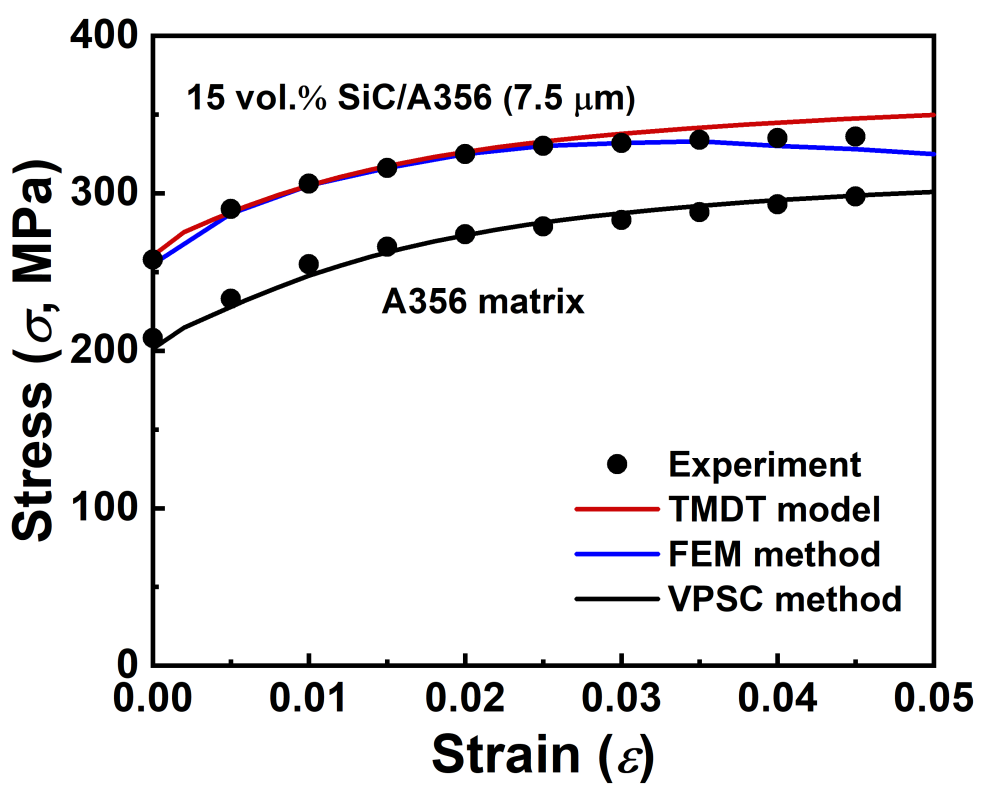


**Supplementary Figure 4.** The mechanical properties of SiC/A356 composites calculated by the FEM and VPSC methods, as well the VPSC-TMDT model.


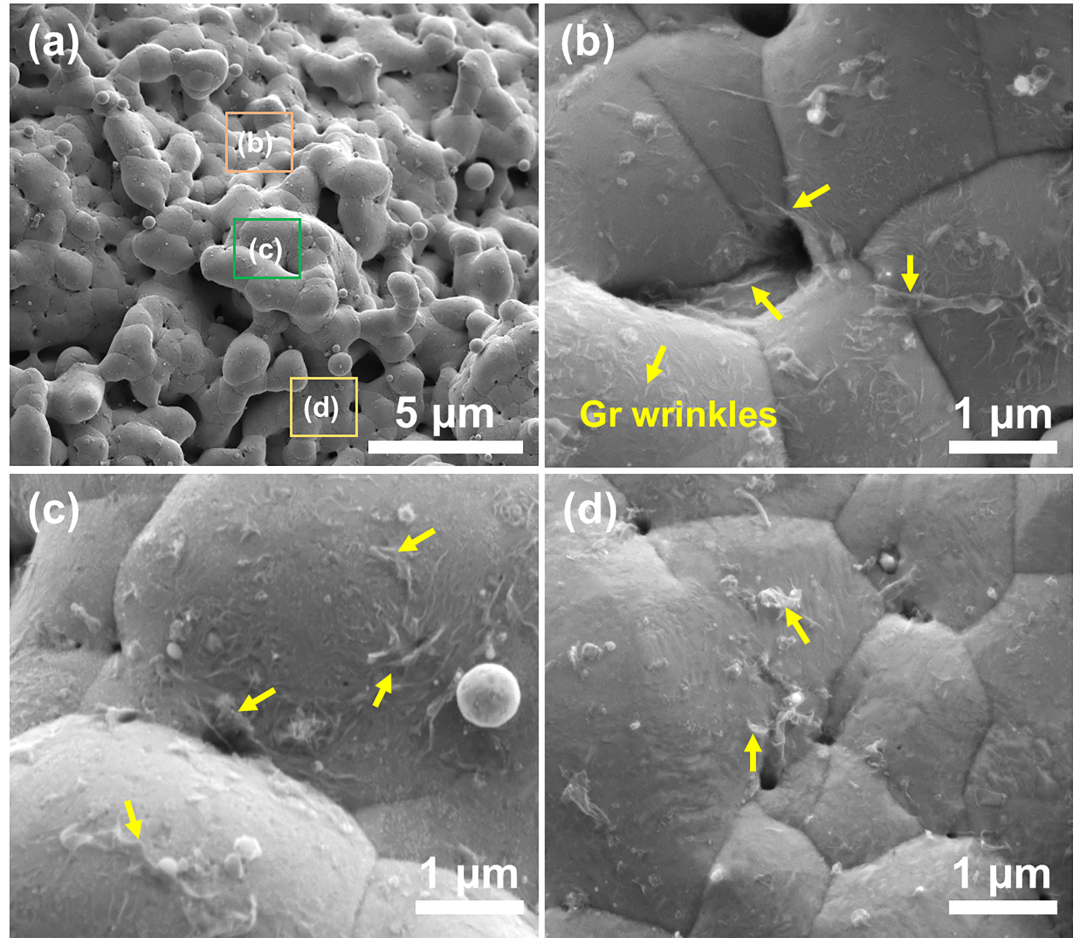


**Supplementary Figure 5. SEM images of the Gr/Cu composite powders.** The results suggesting that the Gr is uniformly distributed within the Gr/Cu composite powders. The Gr is judged by its wrinkle morphology and is indicated by the yellow arrows. (b-d) are the magnified areas corresponding to the solid frame areas in (a).

**Supplementary Table 1.** Material parameters of the SiC and A356 matrix**.**

| **Materials** | $\mathbf{v}$ | $\boldsymbol{\lambda}$ | $\boldsymbol{\mu(GPa)}$ | **E (**$\mathbf{GPa}$**)** | $\boldsymbol{\tau}_{\mathbf{ym}}\mathbf{(kg/}\mathbf{mm}^{\mathbf{2}}\mathbf{)}$ | $\boldsymbol{\gamma}_{\mathbf{T}}\mathbf{(mJ/}\mathbf{m}^{\mathbf{2}}\mathbf{)}$ |
| --- | --- | --- | --- | --- | --- | --- |
| SiC | 0.17 | 132 | 257 | 427 | — | — |
| A356 | 0.33 | 56 | 28.5 | 76 | 18 | 75 |

**Supplementary Table 2.** Material parameters of the Gr and Cu matrix.

| **Materials** | $\mathbf{v}$ | $\boldsymbol{\lambda}$ | $\boldsymbol{\mu(GPa)}$ | **E (**$\mathbf{GPa}$**)** | $\boldsymbol{\tau}_{\mathbf{ym}}\mathbf{(kg/}\mathbf{mm}^{\mathbf{2}}\mathbf{)}$ | $\boldsymbol{\gamma}_{\mathbf{T}}\mathbf{(mJ/}\mathbf{m}^{\mathbf{2}}\mathbf{)}$ | $\mathbf{d}_{\mathbf{t}}\mathbf{(nm)}$ | $\mathbf{t (nm)}$ |
| --- | --- | --- | --- | --- | --- | --- | --- | --- |
| Gr | 0.186 | 239 | 424 | 1000 | — | — | — | — |
| Cu | 0.33 | 69 | 46 | 120 | 30 | 24 | 140 | 9 |

**Supplementary Note 1**

**Constitutive relation of MMCs considering thermal mismatch dislocation in FEM method.** As shown in Fig. 5, the element in FEM method is divided into three different regions: spherical particle reinforcement (region A), plastic accommodating region (region B) and non-affected region (region C). The range of region B is denoted as $r_{p}$. The flow stress of region B ($\sigma_{flow}^{(B)}$) is calculated based on the thermal mismatch dislocation density ($\rho^{CTE}$) and geometrical mismatch dislocation density ($\rho^{GM}$)^1^:

$\sigma_{flow}^{(B)}=\sqrt{{(\sigma_{y}+\alpha\mu b\sqrt{\rho^{CTE}})}^{2}+M\beta\mu b\rho^{GM}}$ (1)

where $\sigma_{y}$ is the yield stress of matrix, $\alpha$ is the Taylor’s coefficient for matrix, $\mu$ is the shear modulus, $b$ is the Burgers vector of the slip system, *M* is the Taylor factor and $\beta$ is an empirical coefficient. The flow stress of region C ($\sigma_{flow}^{(C)}$) is determined by the $\rho^{GM}$:

$\sigma_{flow}^{(C)}=\sqrt{{(\sigma_{y})}^{2}+M\beta\mu b\rho^{GM}}$ (2)

**Supplementary Note 2**

**Constitutive relation of polycrystal metal in VPSC method.** In the VPSC method, each grain is regarded as an ellipsoidal visco-plastic inclusion embedded in an effective visco-plastic medium. This medium represents the aggregate of all textured metal grain. The stress and strain rate are uniform within the ellipsoidal grain. Then the strain rate of grain ($\dot{\varepsilon}_{ij}^{g}$) is given by the sum of shear rate $\dot{\gamma}^{s}$ over the active systems^2^:

$\dot{\varepsilon}_{ij}^{g} = \sum_{s} \dot{\gamma}^{s}m_{ij}^{s}=\dot{\gamma_{0}}\sum_{s} m_{ij}^{s}\left( \frac{m_{pq}^{s}\sigma_{pq}^{g}}{\tau_{C}^{s}} \right)^{n}sgn\left( m_{pq}^{s}\sigma_{pq}^{g} \right)$ (3)

where $m_{ij}^{s}$is the Schmid tensor of the slip system *s*, $\dot{\gamma_{0}}$ is a reference shear rate, *n* is the strain rate sensitivity, $\sigma_{pq}^{g}$ is the stress applied on the grain, $\tau_{C}^{s}$ is the critical resolved shear stress (CRSS) of the slip system *s*, and it is followed by defect hardening laws^3^.

Besides, the interaction between the grain and medium can be expressed as^4^: $\dot{\varepsilon}_{ij}^{g}-{\bar{\dot{\varepsilon}}}_{ij}=-\tilde{M}_{ijpq}(\sigma_{pq}^{g}-\bar{\sigma}_{pq})$ (4)

where ${\bar{\dot{\varepsilon}}}_{ij}$ are the strain rate of the medium, $\tilde{M}_{ijkl}$ is interaction tensors between the grain and medium, $\bar{\sigma}_{kl}$ are the stress of medium.

**Supplementary Note 3**

**Calculations of the** **specific parameters for the thermal mismatch dislocations and twins.** First, the distribution range ($r_{p}$) and density ($\rho^{CTE}$) of the thermal mismatch dislocations were calculated according to the spherical dislocation punching model^2,3^. The relationship between the $r_{p}$ and the radius ($a$) of the reinforcement particle is described by:

$\left( \frac{r_{p}}{a} \right)^{3} = \frac{B\left( 1-2Pf \right)+\sqrt{B^{2}\left( 1 - 2Pf \right)^{2}+16\left( {\tau_{ym}}/{\mu_{m}} \right)PB}}{4\left( {\tau_{ym}}/{\mu_{m}} \right)}$ (5)

$B= \frac{\left( 1+\nu_{m} \right)\left| \varepsilon_{m} \right|}{\left( 1-\nu_{m} \right)}$ (6)

$P = \frac{2\left( 1-2\nu_{m} \right)\left( 3\bar{\lambda}+2\bar{\mu} \right)}{\left( 1-v_{m} \right)\left\{ \left( 1-f \right)\left( 3\bar{\lambda}+2\bar{\mu} \right)\left( \frac{1+\nu_{m}}{1-\nu_{m}} \right)+3\left[ f\left( 3\lambda_{p}+2\mu_{p} \right)+\left( 1-f \right)\left( 3\lambda_{m}+2\mu_{m} \right) \right] \right\}}$ (7)

The matrix and particle are marked as by the subscripts *m* and *p*, respectively. In Eq. (5-7), $f$ is the volume fraction of the reinforcement, $\tau_{ym}$ is the shear yield strength of the matrix, $\mu$ is the shear modulus, $\nu$ is the Poisson’s ratio, $\varepsilon_{m}$ is the thermal mismatch strain, and $\bar{\lambda}$ and $\bar{\mu}$ are the differences in the Lame constants. Regarding the $\rho^{CTE}$ calculation, it is expressed as^3,4^:

$\rho^{CTE}=\frac{6\sqrt{2}\varepsilon_{m}}{b}\frac{a^{2}}{{r_{p}}^{3}-a^{3}}$ (8)

where $b$ is the Burgers vector of the slip systems.

Then, the thickness ($d_{t}$) and spacing (*t*) of the thermal mismatch twins are deduced through the energy minimization method^5,6^. The total energy ($E_{t}$) of the thermal misfit twins can be obtained by:

$E_{t}=E_{s,twin}+E_{int,twin,prep}+E_{int,twin,m}+E_{m}+\tilde{\Gamma}_{p}/t$ (9)

where $E_{s,twin}$, $E_{int,twin,prep}$, $E_{int,twin,m}$, $E_{m}$ and $\tilde{\Gamma}_{p}$ are the self-energy, the perpendicular twin interaction energy, the interaction energy between twins and the mismatch stress, the mismatch strain energy and the total twin-boundary energy, respectively. Concerning the $d_{t}$ and $t$ of the equilibrium twins, they are calculated based on the values of $\mu$, $\nu$, $\varepsilon_{m}$, the average spacing of reinforcement ($D$) and the twin boundary energy ($\gamma_{T}$) through^5^:

$\frac{\partial E_{t}\left( d_{t},t,\varepsilon_{m},D \right)}{\partial t}=0$ (10)

$\frac{\partial E_{t}\left( d_{t},t,\varepsilon_{m},D \right)}{\partial d_{t}}= 0$ (11)

$D=\sqrt[3]{\frac{16}{3f}}a$ (12)

Besides, due to the average percentage ($P_{t}$) of the strain relaxation keeps unchanged under the same $\varepsilon_{m}$, therefore, the relationship between $d_{t}$ and $t$ can be presented by^5^:

$\frac{t}{d_{t}}=\frac{b_{1}}{P_{t}\varepsilon_{m}}$ (13)

where $b_{1}$ is the Burgers vector component of partial dislocations.

**Supplementary Note 4**

**Preparation of the Gr/Cu composites.** First, 20 g Cu powders were added into 300 mL 0.5 wt% Poly methyl methacrylate (PMMA) anisole solution, and stirred for 12 h. Then, the mixture was centrifuged at 1000 rpm for 60 min. After that, the PMMA coated Cu powders were dried in a vacuum oven at 343 K for 5 h to remove the solvent. The dried PMMA/Cu powders were then placed in a tube furnace for the Gr synthesis. In this process, the Gr growth temperature and time under atmospheric pressure were 930 ^o^C and 1 h, and the gas flow of hydrogen (H_2_) and argon (Ar) were 10 and 500 sccm, respectively. The as-obtained Gr/Cu composite powders were pre-compacted in a graphite die, and were consolidated by spark plasma sintering at 600℃ for 10 min with a pressure of 50 MPa. In order to exclude the effect of residual stress on the defect characterizations, all the as-prepared Gr/Cu composites were annealed at 300 ^o^C for 3 h.

**Characterizations.** Microstructures of the Gr/Cu nanocomposites were characterized using a field-emission scanning electron microscopy (SEM, S-4800, HITACHI) and TEM at 200kV (JEM 2100F, JEOL).

**Supplementary References:**

1 Shao, J. C., Xiao, B. L., Wang, Q. Z., Ma, Z. Y. & Yang, K. An enhanced FEM model for particle size dependent flow strengthening and interface damage in particle reinforced metal matrix composites. *Compos Sci Technol* **71**, 39-45 (2011).

2 Kabirian, F., Khan, A. S. & Gnaupel-Herlod, T. Visco-plastic modeling of mechanical responses and texture evolution in extruded AZ31 magnesium alloy for various loading conditions. Int. J. Plast. 68, 1-20 (2015).

3 Beyerlein, I. J. & Tomé, C. N. A dislocation-based constitutive law for pure Zr including temperature effects. Int. J. Plast. 24, 867-895 (2008).

4 Kitayama, K., Tomé, C., Rauch, E. F., Gracio, J. J. & Barlat, F. A crystallographic dislocation model for describing hardening of polycrystals during strain path changes. Application to low carbon steels. Int. J. Plast. 46, 54-69 (2013).

5 Shibata, S., Taya, M., Mori, T. & Mura, T. Dislocation punching from spherical inclusions in a metal matrix composite. *Acta Metall. Mater* **40**, 3141–3148 (1992).

6 Dunand, D. C. & Mortensen, A. On plastic relaxation of thermal stresses in reinforced metals. *Acta Metall. Mater* **39**, 127-139 (1991).

7 Taya, M. L., K. E Lloyd, D. J. Strengthening of a particulate metal matrix composite by quenching. *Acta Metall. Mater* **39**, 77-87 (1991).

8 Zhang, Y. S., Liu, L. L. & Zhang, T. Y. Strain relaxation in heteroepitaxial films by misfit twinning: II. Equilibrium morphology. *J. Appl. Phys.* **101**, 13 (2007).

9 Liu, L. L., Zhang, Y. S. & Zhang, T. Y. Strain relaxation in heteroepitaxial films by misfit twinning. I. Critical thickness. *J. Appl. Phys.* **101**, 12 (2007).
